# Impact of associated injuries in the Floating knee: A retrospective study

**DOI:** 10.1186/1471-2474-10-7

**Published:** 2009-01-14

**Authors:** Ulfin Rethnam, Rajam S Yesupalan, Rajagopalan Nair

**Affiliations:** 1 Department of Orthopaedics, Glan Clwyd Hospital, Bodelwyddan, UK; 2 Department of Accident & Emergency, Glan Clwyd Hospital, Bodelwyddan, UK; 3 Department of Orthopaedics, St John's Medical College, Bangalore, India

## Abstract

**Background:**

Floating knee injuries are usually associated with other significant injuries. Do these injuries have implications on the management of the floating knee and the final outcome of patients? Our study aims to assess the implications of associated injuries in the management and final outcome of floating knee.

**Methods:**

29 patients with floating knees were assessed in our institution. A retrospective analysis of medical records and radiographs were done and all associated injuries were identified. The impact of associated injuries on delay in initial surgical management, delay in rehabilitation & final outcome of the floating knee were assessed.

**Results:**

38 associated injuries were noted. 7 were associated with ipsilateral knee injuries. Lower limb injuries were most commonly associated with the floating knee. Patients with some associated injuries had a delay in surgical management and others a delay in post-operative rehabilitation. Knee ligament and vascular injuries were associated with poor outcome.

**Conclusion:**

The associated injuries were quite frequent with the floating knee. Some of the associated injuries caused a delay in surgical management and post-operative rehabilitation. In assessment of the final outcome, patients with associated knee and vascular injuries had a poor prognosis. Majority of the patients with associated injuries had a good or excellent outcome.

## Background

Floating knee injuries (ipsilateral fractures of the femur and tibia) are always due to high energy trauma. The force required to fracture two of the strongest bones in the body is immense. Not surprisingly, these injuries are associated with other injuries (bony and soft tissue). These patients are usually haemodynamically unstable and need close monitoring and resuscitation during the initial period of following injury.

The need for a thorough secondary survey cannot be overemphasised. The grossly deformed limb that one encounters in the floating knee can act as a major "distracting factor" and it is not unusual to miss other significant injuries. Do these injuries have a bearing on the management on the floating knee? Most studies on the floating knee concentrate on their treatment, with just a mention of the associated injuries. [1-7]

Our study aims to assess the impact of associated injuries in the management and final outcome of the floating knee.

## Methods

This study was conducted over a 3 year period in a tertiary care teaching hospital after approval of the Hospital Research & Ethics Committee (St. John's Medical College).

This was a retrospective analysis of associated injuries in patients with floating knee injuries. The study included all patients with surgically treated ipsilateral fractures of the femur and tibia encountered during the study period. Data was collected from the medical records of patients and the bony injuries where confirmed on radiographs taken. The data collected included the type of associated injury, its treatment, delay in surgical management of the floating knee due to associated injuries, delay in rehabilitation and their impact on the final outcome.

After primary survey, resuscitation and splinting of the affected limb, a thorough secondary survey was performed to identify any associated injuries. This involved examining the patient from head to toe looking for any evidence of fractures or other injuries. Relevant radiographs were done for a suspected associated injury. Open fractures were managed according to the departmental protocol (wound toilet, antibiotics and urgent surgical stabilisation of fractures). Once the associated injuries were identified, treatment was planned together with management of the floating knee.

The floating knee injuries encountered were classified according to the Blake and McBryde classification. [8] (Table 1) This classification system included all varieties of floating knee injuries including articular fractures around the knee, fractures of the hip and ankle. This was therefore a comprehensive classification as compared to the other classification systems that exist for the floating knee. [9,10] After resuscitation, the floating knee fractures were managed surgically. The identified associated injuries were treated appropriately under the same anaesthesia. Patients were assessed after clinical and radiological union of the fractures. The end result was assessed by the Karlstrom criteria. (Table 2) [11]

**Table 1:** Blake and McBryde classification for Floating Knee injuries

Type 1 – True Floating Knee	The knee joint is isolated completely and not involved, with either shaft fractured
Type 2 – Variant Floating knee	Involves one or more joints with either shaft fractured.

Type 2A	The knee joint alone is involved

Type 2B	Involves the hip or ankle joints

**Table 2:** Karlstrom criteria for functional assessment after management of floating knee injuries

Criterion	Excellent	Good	Acceptable	Poor
Symptoms from thigh or leg	None	Intermittent slight symptoms	More severe symptom impairing function	Considerable functional impairment: pain at rest
Symptoms from knee or ankle joint	None	Same as above	Same as above	Same as above
Walking ability	Unimpaired	Same as above	Walking distance restricted	Uses cane, crutch or other support
Work and sports	Same as before	Given up sport; work same as before	Change to less strenuous work	Permanent disability
Angulation, rotational deformity or both	0	< 10 degrees	10 – 20 degrees	> 20 degrees
Shortening	0	< 1 centimetre	1 – 3 centimetres	> 3 centimetres
Restricted joint mobility	0	< 10 degrees at ankle; < 20 degrees at hip, knee or both	10 – 20 degrees at ankle; 20 – 40 degrees at hip, knee or both	> 20 degrees at ankle; > 40 degrees at hip, knee or both

## Results

29 patients with floating knees were included in the study. There were 27 males and 2 females. The mean age was 28 years (Range 18 – 56). Road traffic accidents accounted for 27 patients while 2 patients sustained their injuries after a fall from height. The right side was involved in 19 patients and the left side in 10 patients. There were 21 Type 1 floating knees, 4 Type 2A injuries and 5 Type 2B injuries (Blake and McBryde classification, Table 1). None of the patients had any previous significant medical problems such as cardiovascular or pulmonary disease, previous deep vein thrombosis, pulmonary embolism increasing operative risk or causing delay in initial surgical management.

Most of the floating knee injuries were treated with antegrade intramedullary nailing for both the femur and tibia (20/29 patients). Other modes of treatment included dynamic hip screw fixation, dynamic condylar screw fixation, tibial buttress plate fixation and external fixation. The femoral fracture was stabilised initially followed by the tibial fracture. Surgical stabilisation of associated fractures was performed under the same anaesthesia. Chemical thromboprophylaxis with enoxaparin was started on all patients at time of admission and this was continued post-operatively. The post operative regime was to start knee range of movement exercises and mobilisation partial weight bearing using crutches on the second post operative day by physiotherapists. Continuous passive motion was used daily for knee mobilisation till satisfactory knee movements were achieved. After union of the fractures, assessment by the Karlstrom criteria revealed the following results; excellent – 15, good – 10, acceptable – 1, poor – 3. The mean follow up was 24.2 months (Range 16 – 28 months).

38 associated injuries were noted in the 29 patients. Only 3 of the 29 patients had isolated floating knee injuries. The associated injuries ranged from head injury to metatarsal fractures. (Table 3)

**Table 3:** Associated injuries with Floating knee and their management

Associated injury	Patients	Intervention
Patellar fractures	3	Open reduction internal fixation
Knee ligament injuries	4	Ligament repair, medial meniscectomy
Clavicle fractures	4	Conservative
Femoral fractures (opposite)	3	Intramedullary nailing
Femoral artery injury	1	Femoro-popliteal bypass graft
Humeral shaft fractures	4	Open reduction internal fixation
Head injury	3	Conservative
Rib fractures	1	Conservative
Haemo-pneumothorax	2	Chest drain insertion
Forearm bones fractures	1	Open reduction internal fixation
Contralateral tibial fractures	4	Intramedullary nailing
Metatarsal fractures	4	Conservative
Fat embolism	3	Mechanical ventilation
Radial nerve palsy	1	Conservative

### Head and neck injuries

3 patients sustained head injuries for which a CT scan of the brain was done. The initial assessment included a neurological assessment, pupil size and the Glasgow Coma Scale (GCS). A clinical and radiological assessment of the neck was done to look for cervical spine injuries. None of these patients had intracranial bleeds or haematomas that needed intervention by the neurosurgeons. All of these patients were diagnosed to have cerebral concussion and had supplemental oxygen till the post operative period. Surgical fixation of the fractures was done once they were neurologically stable with a GCS of 15. 2 head injured patients had contralateral tibia & femur fractures respectively. (Table 4) There was a mean delay in surgical management of the floating knee due to head injury of 2 days (Range 1–3 days). There was no delay in rehabilitation due to the head injury and all the three patients had an excellent final outcome when assessed using the Karlstrom criteria. (Table 4) None of the patients in the study sustained neck injuries.

**Table 4:** Implications of associated injuries in the floating knee

Patient	Associated injury	Injury Severity Score	Delay in primary surgery	Surgical duration (Min)	Delay in rehabilitation	Final outcome (Karlstrom)
1	None	9	0	140	Nil	Excellent
2	Cerebral concussion	18	2 days	120	Nil	Excellent
3	Clavicle, Fat embolism	9	8 days	110	Nil	Excellent
4	Patella	9	0	180	4 weeks	Good
5	Contralateral femur	9	0	200	2 weeks	Good
6	Anterior Cruciate tear	9	0	210	4 weeks	Poor
7	Clavicle, Humerus, Forearm, Metatarsal	9	0	210	4 weeks	Good
8	Medial meniscus injury	9	0	180	Nil	Good
9	None	9	0	120	Nil	Excellent
10	Contralateral tibia	9	0	150	2 weeks	Excellent
11	Humerus	9	0	170	4 weeks	Excellent
12	Fat embolism	9	11 days	110	Nil	Excellent
13	Clavicle, Haemo-pneumothorax	25	0	130	Nil	Excellent
14	Metatarsal	9	0	120	Nil	Excellent
15	Humerus, Radial nerve	9	0	180	4 weeks	Good
16	Contralateral tibia, Fat embolism	9	9 days	160	2 weeks	Good
17	Contralateral femur	9	0	170	2 weeks	Good
18	Posterior Cruciate tear	9	0	200	4 weeks	Poor
19	Clavicle, Rib, Haemo-pneumothorax	25	0	120	2 weeks	Excellent
20	Patella, Metatarsal	9	0	160	4 weeks	Good
21	None	9	0	110	Nil	Excellent
22	Contralateral tibia, Cerebral concussion	18	3	170	2 weeks	Excellent
23	Humerus	9	0	180	3 weeks	Excellent
24	Femoral artery injury	9	0	260	3 weeks	Poor
25	Contralateral tibia	9	0	180	2 weeks	Good
26	Anterior Cruciate tear	9	0	200	4 weeks	Acceptable
27	Metatarsal	9	0	130	Nil	Excellent
28	Contralateral femur, concussion	18	1	180	2 weeks	Excellent
29	Patella	9	0	160	4 weeks	Good

### Fat embolism

3 patients were diagnosed to have fat embolism within 48 hours of admission. (Table 4) The diagnosis was made if the patients had pyrexia, tachycardia, tachypnoea and had altered sensorium after admission. Blood gases confirmed hypoxia in these patients who were managed in the intensive care unit with mechanical ventilation. Surgical management of the fractures were delayed by a mean 9 days (Range 8–11 days)in these patients. Fat embolism did not cause any delay in rehabilitation of the patients after surgery. 2 patients had excellent results and one patient a good result on assessment of the final outcome.

### Chest & abdomen injuries

2 patients were clinically and radiologically diagnosed to have haemopneumothorax that needed tube thoracostomy insertion. (Table 4) The chest drain was inserted in the Accident & Emergency and removed when the haemopnemothorax had radiologically reduced and the patient was symptomatically better. 1 patient had multiple rib fractures which delayed the post operative rehabilitation by 2 weeks. The chest injuries did not seem to have an impact on the final outcome as both these patients had excellent results. All patients had clinical evaluation of the abdomen on arrival to the hospital and none had signs of any intra-abdominal injuries.

### Associated upper limb injuries

10 injuries were encountered in 7 patients. Most of the injuries were fractures with 1 patient sustaining a radial nerve injury. The clavicle and humerus was the most common upper limb bones fractured. The clavicle fractures were managed conservatively while all humerus fractures were surgically fixed (Dynamic compression plating). In the patient with radial nerve palsy, the radial nerve was explored during fixation of the humerus and found to be contused therefore managed conservatively. The clavicle fractures did not have an impact on time for rehabilitation or the final outcome as all these patients had either excellent or good results. (Table 4) The average surgical duration was 185 +/- 17.3 minutes (Range 170 – 210) when upper limb fractures were fixed along with the floating knee as compared to 121 +/- 9.9 minutes (Range 110 – 140) with fixation of isolated floating knee. There was a mean delay of 3.75 weeks (Range 3 – 4 weeks) for post operative rehabilitation with upper limb injuries that were surgically fixed (Humerus and forearm). Upper limb injuries did not have a negative impact on the final outcome as all patients had an excellent to good result. (Table 4)

### Associated lower limb injuries

16 patients with 18 lower limb injuries were encountered. Most injuries were fractures. (Table 4) Contralateral tibia and metatarsal fractures were the most common associated lower limb fracture (4 patients) with contralateral femur and ipsilateral patella fractures the next common fractures (3 patients). The average surgical duration was 184 +/- 27.4 minutes (Range 150 – 260) when lower limb injuries were fixed along with the floating knee as compared to 121 +/- 9.9 minutes (Range 110 – 140) with fixation of isolated floating knee. The mean delay in rehabilitation was 2.6 weeks (Range 2 – 4 weeks) with lower limb injuries. This delay was more in patients with ipsilateral knee injuries (patella fractures, crucuaite ligament injuries) as compared to foot fractures or contralateral lower limb fractures (tibia and femur). Contralateral femur or tibia fractures did not have a negative impact in the final outcome. (Table 4)

1 patient had a femoral artery injury which was suspected clinically and evaluated by a femoral angiogram. This revealed an intimal injury of the superficial femoral artery that needed a femoro-popliteal bypass graft which was performed by the vascular surgeons after surgical stabilisation of the fractures. Surgical stabilisation of the fractures was done initially to avoid placing stress on the vascular bypass graft during reduction of the fractures. The rehabilitation of this patient was delayed by 3 weeks and the final outcome assessed by the Karlstrom criteria was poor.

### Associated ipsilateral knee injuries

7 patients had ipsilateral knee injuries (3 patellar fractures, 2 anterior cruciate ligament tears, 1 posterior cruciate ligament tear and a bucket handle medial meniscal tear). Knee ligament injuries were diagnosed by clinical assessment by the surgeon after surgical stabilisation of the fractures. Lachman's test and posterior drawer's test were used to clinically assess the anterior and posterior cruciate ligaments respectively. If a knee ligament injury was suspected, a diagnostic arthroscopy was performed under the same anaesthesia and primary ligament repair done. The patellar fracture surgical stabilisation was done under the same anaesthesia. The mean delay in rehabilitation was 4 weeks in patients with ipsilateral knee injuries as these patients were placed in a brace post operatively. There were 2 poor results and an acceptable result in patients with knee ligament injuries. (Table 4)

The complications encountered were knee stiffness in 4 patients, delayed union of tibia in 2 patients and superficial infection in 2 patients. The secondary procedures were manipulation under anaesthesia for knee stiffness, dynamisation in one patient with delayed union. Patients with delayed union needed either dynamisation of the tibial nail or removal of external fixator and functional bracing of the fracture. These fractures went on to unite following these interventions. The superficial infections were related to pin sites of the external fixators which were managed by pin site care and antibiotics. The infection settled with this management.

## Discussion

Floating knee injuries are caused by high energy trauma which can have an effect on other parts of the body. These patients sustain significant and occasionally life threatening associated injuries. Many studies in the literature mention associated injuries like head, chest, abdominal injuries and injuries to other extremities. [12] Injuries to the head, chest and abdomen can be life threatening. The reported mortality rate ranged from 5–15% reflecting the seriousness of associated injuries in the floating knee. [12] There is a higher incidence of neurovascular and soft tissue injury in this injury. Surgical stabilisation of both fractures in the floating knee has been found to have the best outcome results. [5]

As these injuries occur consequent to high velocity trauma, the associated injuries (head injury, chest, vascular injury and other fractures have a significant role in surgical decision making with regards to timing of surgery and sequence of surgery. Any associated medical co-morbidities can worsen the already compromised physiologic reserve especially in the elderly.

Poole GV et al (13) in a comparison study on lower extremity fracture fixation in the head injured patient found that surgical stabilisation of fractures within 24 hours of injury reduced the risk of pulmonary complications (fat embolism, pneumonia and adult respiratory distress syndrome. [13] Cerebral injury has been found to be associated with high risk of pulmonary complications. [14,15] A delay in fracture fixation did not protect the injured brain. There is no data in the literature that the injured brain is at risk for further injury during the surgical procedure provided the patient is not exposed to hypotension and hypoxia. In our study patients with head injury had CT scan to the brain to rule out an intracranial bleed or haematoma. There was a delay in surgery for these patients as they had a fluctuating Glasgow Coma scale and once their GCS had become normal we stabilised the fractures. All patients with fluctuating conscious levels need a CT scan of the brain. If an intracranial haematoma or bleed is diagnosed these patients should be referred to the neurosurgery unit for further management.

Vascular assessment of the affected limb is of utmost importance in detecting any vascular injury. Cakir O et al [16] in their study on the treatment of vascular injuries associated with limb fractures recommended careful assessment of the peripheral pulses by palpation or hand held Doppler. If an arterial injury was suspected, a pre-operative angiogram was done if there was no critical ischaemia to the limb. There has been a well established debate over the sequence of surgical vascular repair and bone stabilisation. [16-20] McHenry et al in their study found no iatrogenic disruption of the vascular repair when bone stabilisation followed vascular repair. [21] The general consensus is that bone stabilisation should precede vascular repair in unstable fractures while in stable fractures vascular repair should be done first to avoid prolonged ischaemia to the limb. In our study there was one patient with a superficial femoral artery injury which was diagnosed by absent distal pulses using a hand held Doppler. This patient was referred to the vascular surgeons who performed an on-table angiogram followed by a femoro-popliteal bypass graft. If a vascular injury is suspected, referral to the vascular surgeons is the norm and assessment of the affected limb by an angiogram is performed. Repair of the vascular injury is best done after surgical stabilisation of the fractures as the floating knee is an unstable injury and manipulation of the fracture after repair of a vessel can put stress on the repair leading to failure of the repair. In our study the patient with vascular injury had a delay in rehabilitation and a poor final outcome. Vascular injuries associated with the floating knee are a poor prognostic indicator and should be assessed and managed with care.

Many studies have documented that early surgical stabilisation of fractures were associated with significant reduction in pulmonary complications such as fat embolism, pneumonia and Adult Respiratory Distress Syndrome. [22,23]

When encountered in the elderly, the diminished physiologic reserve and pre-existing co-morbid conditions may contribute to higher morbidity and mortality following a floating knee injury. The principle here is to manage the patient's co-morbidities (cardio-respiratory, renal etc) till the patient is fit to undergo surgery. Surgery after this has shown to improve the patient's final outcome. [24,25]

If significant abdominal injuries are detected, these take priority over surgical stabilisation of the fractures. Detection of abdominal injuries should be by clinical assessment and ultrasonography. If there was a suspicion of intra-abdominal injury, an urgent CT scan is indicated. In our study, we did not have any patients with significant abdominal trauma, intracranial haematoma or bleed.

Associated ipsilateral knee ligament injuries are common in the floating knee and this is documented in the literature. [6,12,26] These can be a diagnostic enigma in the floating knee due to difficulty in clinical assessment of the knee in the presence of fractures on both sides of the knee. These injuries are easily missed due to the "distracting" nature of a floating knee injury. Appropriate management of the knee ligament injury is essential for a good outcome after treatment of the floating knee. Szalay MJ et al in their study of 34 floating knees found detectable ligament laxity in 53%. [26] The Lachmann test is almost 100% diagnostic of anterior cruciate ligament tear when performed under anaesthesia. [20,27] Moore et al found that when knee ligament injuries were repaired a better range of motion was achieved in femoral fractures. They recommended surgical stabilisation of the fracture, stress testing of knee ligaments, acute arthroscopy and ligament repair if knee ligament tear was suspected. [20] Other studies have favoured acute repair of knee ligaments. [28]

In all patients we assessed the knee ligaments after surgical stabilisation of both the femoral and tibial fractures under the same anaesthesia. If instability was detected we proceeded to perform a diagnostic arthroscopy and repaired the ligaments. This avoided the need for an MRI assessment of the knee and further anaesthetic exposure for ligament reconstruction. We did not perform an MRI evaluation of the knees as assessment (clinical and arthroscopic) to detect knee ligament injuries was satisfactory. Although an MRI is the gold standard investigation for evaluating knee ligament injuries, with a floating knee performing an MRI prior to surgical stabilisation of the fractures would cause problems in the patient who may be haemodynamically unstable. After surgical stabilisation of the fractures there may be interference artefacts from the metal work, preventing proper visualisation of the knee ligaments. We feel that a clinical assessment under anaesthesia followed by a diagnostic arthroscopy is the best method of assessment of ligament injuries in these patients.

Most studies discuss the treatment options of floating knee in great depth but not much is mentioned about the associated injuries. The impact of associated injuries on the treatment of floating knee has never been discussed. We have tried to assess the importance of associated injuries in the floating knee by this study. Although the incidence of associated injuries were high (38 in 29 patients), assessing the final outcome, in our study only four patients (4/29) had less than a good outcome (3 poor and 1 acceptable). These were three patients with associated knee ligament injuries and one patient with a vascular injury. Some of the associated injuries with the floating knee had an impact in the management of the patient with regards to surgical delay and delay in rehabilitation. Patients who sustained head injuries and fat embolism had a delay in surgical treatment as compared to other associated injuries. This delay in surgical treatment had no impact on the final outcome of these patients. Patients with ipsilateral knee injuries (Patella fractures, cruciate ligament injuries) and upper limb fractures (humerus & forearm) tended to have an increased delay in rehabilitation as compared to patients with contralateral lower limb fractures (tibia, femur) and those with chest injuries (rib fractures, haemo-pnemothorax).

The limitation of our study is the small sample size. Even in this small sample the total number of associated injuries was high (38 in 29 patients). Despite this high incidence of associated injuries, the number of injuries for each floating knee was small and this is another limitation of our study as recommendations for treatment are on this small sample.

We recommend

1. Thorough secondary survey to detect associated injuries such as chest, abdomen, vascular injury, head injury and other fractures.

2. Life threatening injuries (Haemothorax, pneumothorax, intraabdominal injury, intracranial bleed or haematoma) to be managed as a priority.

3. Early stabilisation of the fractures by 2 teams to reduce the surgical duration.

4. Knee assessment to detect ligament injuries after stabilisation of the fractures.

5. Management of associated fractures under the same anaesthesia.

6. Post-operative monitoring to detect development of fat embolism.

7. Early institution of aggressive physiotherapy to regain good function.

## Conclusion

There is a high incidence of associated injuries with the floating knee. Most patients with associated injuries with the floating knee had an excellent or good outcome. Despite this, associated injuries are important with regards to planning of management. Once other injuries have been identified, a plan has to be made on the type of management. Adhering to a management protocol should give a better final outcome in patients with this severe injury.

## Competing interests

The authors declare that they have no competing interests.

## Authors' contributions

UR was involved in conducting the study, collecting patient details, reviewing the literature, drafting the manuscript and proof read the final manuscript. RSY was involved in reviewing the literature and proof read the manuscript. RN is the senior author who supervised the study and was responsible for final proof reading of the article.

## Pre-publication history

The pre-publication history for this paper can be accessed here:



## References

[B1] Dwyer AJ, Paul R, Mam MK, Kumar A, Gosselin RA (2005). Floating knee injuries: long-term results of four treatment methods. Int Orthop.

[B2] Fraser RD, Hunter GA, Wadell JP (1978). Ipsilateral fracture of the femur and tibia. J Bone and Joint surgery.

[B3] Hee HT, Wong HP, Low YP, Myers L (2001). Predictors of outcome of floating knee injuries in adults: 89 patients followed for 2–12 years. Acta Orthop Scand.

[B4] Hojer H, Gillquist J, Liljedahl SO (1977). Combined fractures of the femoral and tibial shafts in the same limb. Injury.

[B5] Lundy DW, Johnson KD (2001). "Floating knee" injuries: ipsilateral fractures of the femur and tibia. J Am Acad Orthop Surg.

[B6] Paul GR, Sawka MW, Whitelaw GP (1990). Fractures of the ipsilateral femur and tibia: emphasis on intra-articular and soft tissue injury. J Orthop Trauma.

[B7] Veith RG, Winquist RA, Hansen ST (1984). Ipsilateral fractures of the femur and tibia. A report of fifty-seven consecutive cases. J Bone Joint Surg Am.

[B8] Blake R, McBryde A (1975). The floating knee: Ipsilateral fracture of the tibia and femur. South Med J.

[B9] Fraser RD, Hunter GA, Wadell JP (1978). Ipsilateral fracture of the femur and tibia. J Bone Joint Surg Br.

[B10] Letts M, Vincent N, Gouw G (1986). The "floating knee" in children. J Bone Joint Surg Br.

[B11] Goran K, Olerud S (1977). Ipsilateral fracture of the femur and tibia. J Bone and Joint surgery Am.

[B12] van Raay JJ, Raaymakers EL, Dupree HW (1991). Knee ligament injuries combined with ipsilateral tibial and femoral diaphyseal fractures: the "floating knee". Arch Orthop Trauma Surg.

[B13] Poole GV, Miller JD, Agnew SG, Griswold JA (1992). Lower extremity fracture fixation in head-injured patients. J Trauma.

[B14] Demling R, Riessen R (1990). Pulmonary dysfunction after cerebral injury. Crit Care Med.

[B15] Helling TS, Evans LL, Fowler DL, Hays LV, Kennedy FR (1988). Infectious complications in patients with severe head injury. J Trauma.

[B16] Cakir O, Subasi M, Erdem K, Eren N (2005). Treatment of vascular injuries associated with limb fractures. Ann R Coll Surg Engl.

[B17] Ashworth EM, Dalsing MC, Glover JL, Reilly MK (1988). Lower extremity vascular trauma: a comprehensive, aggressive approach. J Trauma.

[B18] Bishara RA, Pasch AR, Lim LT, Meyer JP, Schuler JJ, Hall RF, Flanigan DP (1986). Improved results in the treatment of civilian vascular injuries associated with fractures and dislocations. J Vasc Surg.

[B19] Karavias D, Korovessis P, Filos KS, Siamplis D, Petrocheilos J, Androulakis J (1992). Major vascular lesions associated with orthopaedic injuries. J Orthop Trauma.

[B20] Moore TM, Patzakis MJ, Harvey JP (1988). Ipsilateral diaphyseal femur fractures and knee ligament injuries. Clin Orthop Relat Res.

[B21] McHenry TP, Holcomb JB, Aoki N, Lindsey RW (2002). Fractures with major vascular injuries from gunshot wounds: implications of surgical sequence. J Trauma.

[B22] Behrman SW, Fabian TC, Kudsk KA, Taylor JC (1990). Improved outcome with femur fractures: early vs. delayed fixation. J Trauma.

[B23] Riska EB, Myllynen P (1982). Fat embolism in patients with multiple injuries. J Trauma.

[B24] Davenport HT, Davenport HT (1988). Preparations for anaesthesia for the aged. Anaesthesia in the aged patient.

[B25] Vowles KDJ, Davenport HT (1988). Surgical decision in the aged. Anaesthesia in the aged patient.

[B26] Szalay MJ, Hosking OR, Annear P (1990). Injury of knee ligament associated with ipsilateral femoral shaft fractures and with ipsilateral femoral and tibial shaft fractures. Injury.

[B27] Jonsson T, Althoff B, Peterson L, Renstrom P (1982). Clinical diagnosis of ruptures of the anterior cruciate ligament: a comparative study of the Lachman test and the anterior drawer sign. Am J Sports Med.

[B28] Walling AK, Seradge H, Spiegel PG (1982). Injuries to the knee ligaments with fractures of the femur. J Bone Joint Surg Am.

